# P02.157. Dose-response of spinal manipulation for low back pain: outside care outcomes from a randomized clinical trial

**DOI:** 10.1186/1472-6882-12-S1-P213

**Published:** 2012-06-12

**Authors:** D Vavrek, M Haas, D Peterson

**Affiliations:** 1 University of Western States, Portland, USA

## Purpose

To assess amounts of outside care amongst participants who participated in a randomized clinical dose trial assessing Spinal Manipulative Therapy (SMT) for chronic low back pain (cLBP).

## Methods

Four hundred participants with cLBP were randomized to 4 doses (n = 100/group). Participants were seen three times per week for six weeks, receiving 0, 6, 12, or 18 sessions of SMT, and a light massage control on visits without manipulation. Data were collected until 52 weeks after randomization. Outside care outcomes included prescription and non-prescription use, and treatments outside of the study including the following healthcare providers: chiropractor, primary care physician, surgeon, neurologist, psychiatrist, naturopath/homeopath, nurse practitioner, acupuncturist, physical therapist / occupational therapist, or massage therapist. Preliminary analysis used the chi-square test or Fisher’s exact test of categorized data as appropriate.

## Results

Abstinence from prescription use for prevention of cLBP, within the past four weeks, was greater than 75% of participants using none at any time point. The abstinence from non-prescription medication, within the last four weeks, was greatest in the 18 SMT visit group from week 6 through week 39 follow-up time points with the greatest incidence of non-use at 54% at week six. Seeking care from a chiropractor during the follow-up phase did not differ between groups at any time point with average chiropractor seeking behavior across all four groups reaching a maximum at 52 weeks of 10.6%. Differences between groups seeking outside care from a massage therapist were observed at 18 and 52 weeks, with more SMT associated with less massage care, but this was not consistent across all time points.

## Conclusion

Preliminary analysis of outside care shows that care was balanced across all four dose groups during treatment phases including use of prescription and treatment visits to non-study medical providers and classes. Results from adjusted models will be presented.

